# Cytokeratin 8/18 protects breast cancer cell lines from TRAIL-induced apoptosis

**DOI:** 10.18632/oncotarget.25297

**Published:** 2018-05-01

**Authors:** William P. Bozza, Yaqin Zhang, Baolin Zhang

**Affiliations:** ^1^ Office of Biotechnology Products, Center for Drug Evaluation and Research, Food and Drug Administration, Silver Spring, MD 20993, USA

**Keywords:** death receptor, apoptosis, keratin 8, breast cancer, drug resistance

## Abstract

TNF-related apoptosis inducing ligand (TRAIL) induces apoptosis by engaging its death receptors (DRs) 4 and/or 5 on targeted cells. Clinical attempts to stimulate this apoptotic pathway for cancer therapy, including the use of recombinant human TRAIL (rhTRAIL) or receptor agonistic antibodies, have been underway for over a decade. Unfortunately, these agents have only shown limited therapeutic effects due largely to tumor resistance arising from mechanisms yet to be defined. Here we show that intermediate filament proteins, keratin 8 and keratin 18 (K8/K18), negatively regulate TRAIL induced apoptosis. K8/K18 protein levels are consistently higher in TRAIL-resistant cells compared to TRAIL-sensitive cells in a panel of breast cancer cell lines. Blockade of K8 increased expression of DR5 on the surface of targeted cells and sensitized the cells to TRAIL-induced apoptosis. Conversely, ectopic expression of K8/K18 downregulated DR5 protein expression. K8/K18 appears to negatively regulate apoptosis signaling *via* DR5 in breast cancer cells. Our findings warrant additional studies to determine if K8/K18 could be a predictor of tumor resistance to DR5-targeted therapies.

## INTRODUCTION

Death receptors (DRs) 4 and 5 are cell surface receptors that transmit apoptotic signals initiated by their cognate ligand TNF-related apoptosis inducing ligand (TRAIL). At the molecular level, TRAIL ligation induces DR4/DR5 clustering and subsequently recruits Fas-associated Death Domain protein (FADD) and caspase-8 forming the death-inducing signaling complex (DISC). Proximity-induced autoactivation of caspase-8 within the DISC triggers activation of executioner caspases-3 and -7, which cleave structural proteins ultimately leading to apoptotic cell death [1]. A body of preclinical evidence showed that recombinant human TRAIL preferentially induced apoptosis in cancer cells without harming most normal cells [2, 3]. This unique selectivity led to multiple clinical programs aimed at evaluating the antitumor activities of recombinant human TRAIL and agonistic antibodies against DR4 or DR5 [4]. These agents were shown to have a well-tolerated safety profile in early phases of clinical studies. Unfortunately, their therapeutic effects were limited partly due to tumor resistance arising from various mechanisms such as lack of caspase-8 and caspase-10 expression and upregulation of anti-apoptosis proteins such as c-FLIP, XIAP, and Bcl-2 family member proteins [4]. Recent efforts were shifted to the development of second generation DR5-targeted agents with expected improvement in therapeutic effects [5, 6]. In this context, a better understanding of tumor resistance mechanisms helps identify biomarkers to predict the likelihood of tumor response to DR5-targeted therapies in individual patients. This information can also aid in guiding the selection of combination drugs to achieve better cancer treatment outcomes.

In this study, we identified a novel role of keratins in regulating TRAIL induced apoptosis. Keratins are the major intermediate filament (IF) proteins of the cytoskeleton, where keratin 8 and 18 (K8/K18) pair is preferentially expressed in simple epithelia cells of gastrointestinal tract, liver, pancreas, and mammary gland, from which many carcinomas arise [7-9]. In those cells, K8 and K18 exist as heterodimers and when overexpressed contribute to cancer chemoresistance [10-12] and resistance to apoptosis induced through TNFR-1 [13, 14] or Fas [15-17]. We found that K8/K18 proteins are upregulated in TRAIL-resistant cells when compared to TRAIL-sensitive cells. We also provide evidence that K8/K18 pair negatively regulates DR5 protein stability and surface expression thereby limiting TRAIL induced apoptosis.

## RESULTS

### Keratin 8/18 expression correlates with TRAIL resistance in breast cancer cells

**Figure 1:**
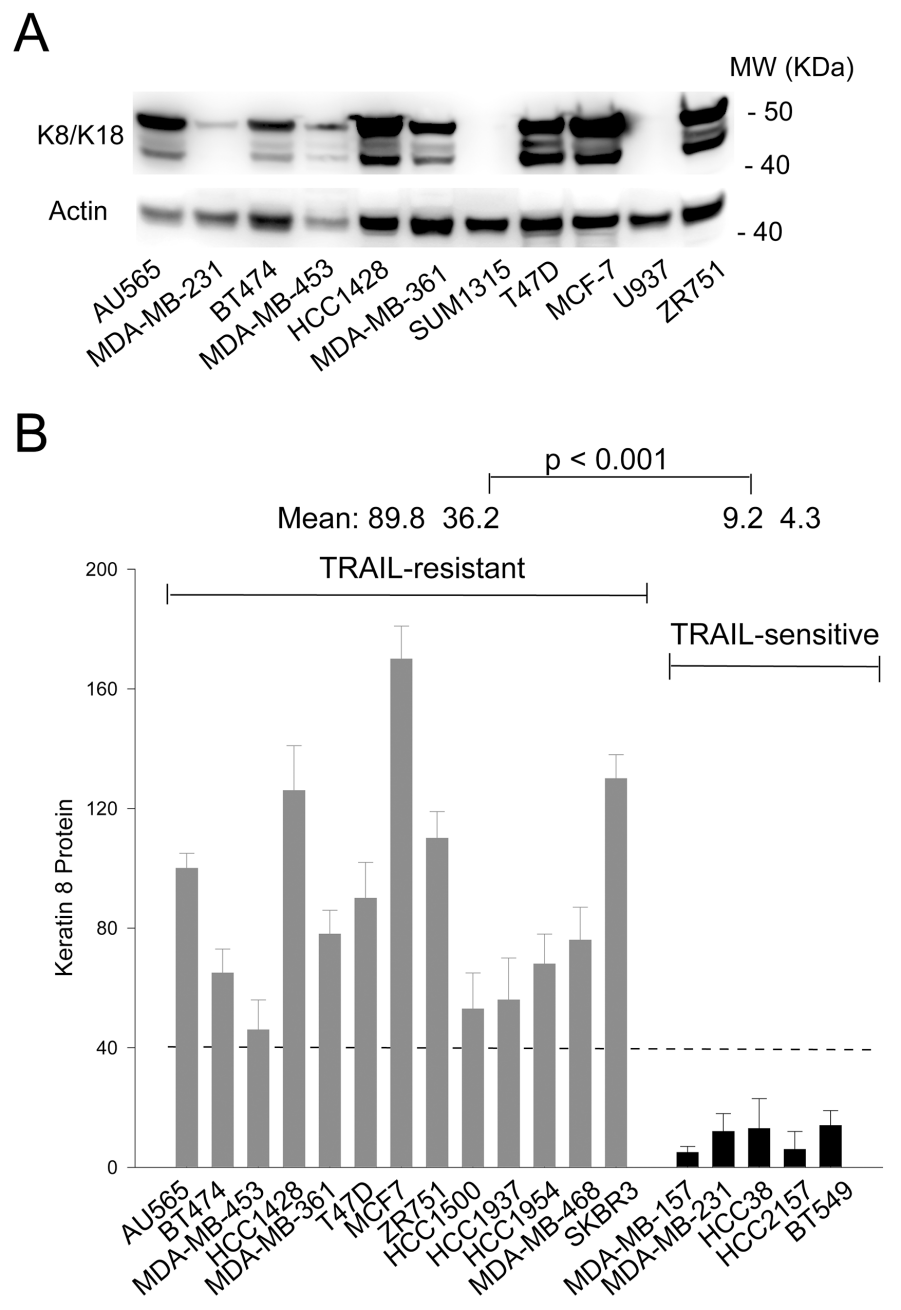
K8/K18 is overexpressed in TRAIL resistant breast cancer cell lines **(A)** Keratin 8/18 protein expression was determined by immunoblot analysis using an antibody recognizing both K8 and K18 in the indicated breast cancer cell lines. **(B)** Relative protein expression levels were determined using densitometry analysis of immunoblots in A and were normalized to the corresponding actin loading controls. Shown are representatives of triplicates.

The intermediate filament protein pair keratin 8 and 18 (K8/K18) have been linked to cellular resistance to several chemotherapeutic drugs [10-12] and to TNF [13, 14] and Fas ligand induced cell killing [15-17]. We hypothesized that K8/K18 might play a role in regulating TRAIL induced apoptosis. To examine this possibility, we measured K8/K18 protein expression in a panel of randomly selected breast cancer cell lines by immunoblotting. Among the cell lines tested, some cells had no detectable levels of K8/K18 and others had saturating amounts under the experimental conditions (Figure 1A). Based on our previous data, this panel of cell lines displayed very different sensitivities to TRAIL killing [18-22]. Notably, K8/K18 protein levels were found to be consistently higher in TRAIL-resistant cells compared to TRAIL-sensitive cells (Figure 1B). These data suggest that K8/K18 pair, when overexpressed, might contribute to TRAIL resistance in breast cancer cells.

### Knockdown of K8 selectively triggers apoptosis *via* DR5

**Figure 2:**
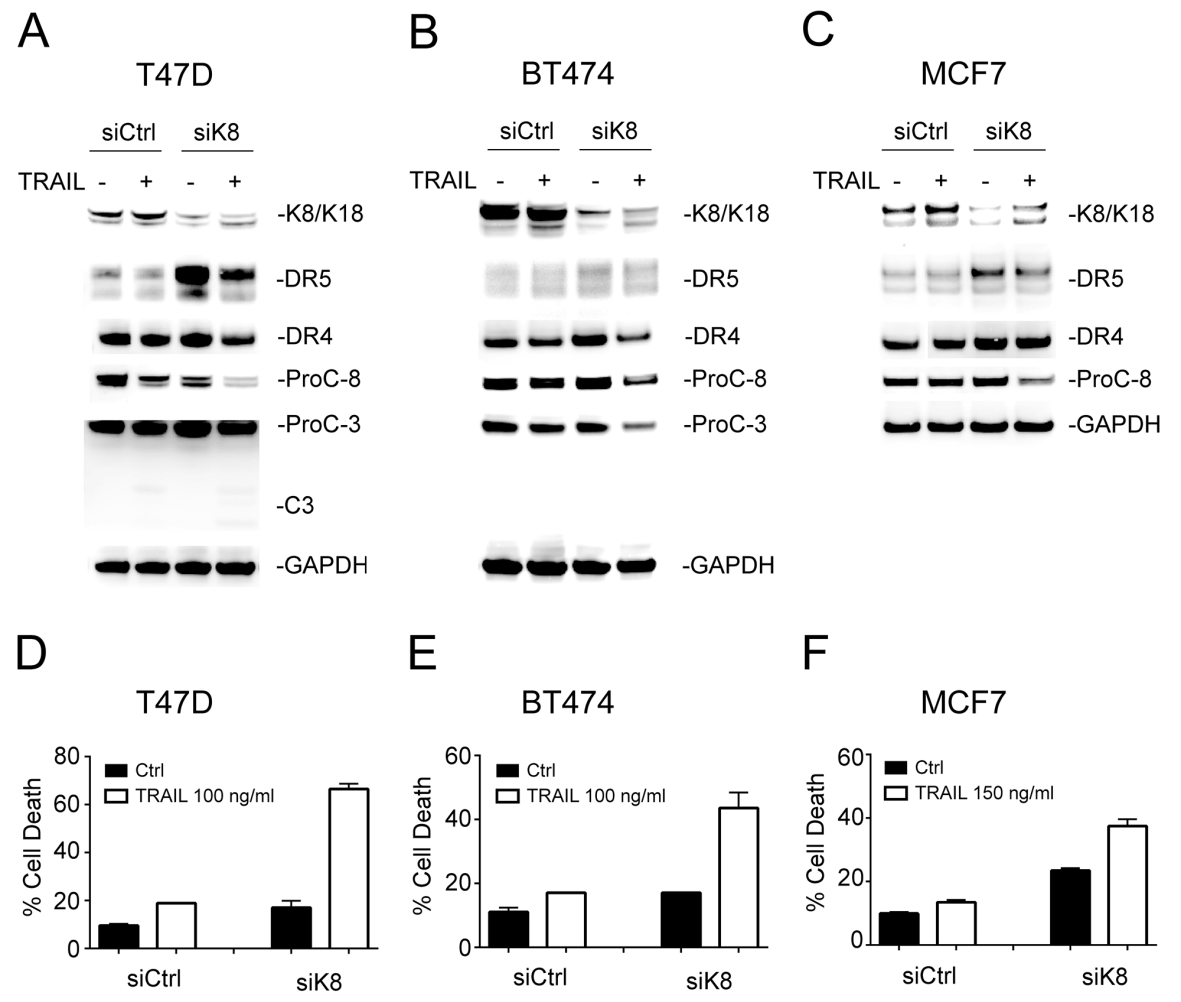
Knockdown of K8 enhances TRAIL induced apoptosis **(A-C)** Cells were transfected with a control siRNA or siRNA specific to *KRT8* transcript for 72 hours, followed by TRAIL stimulation (100 ng/ml [T47D and BT474] or 150 ng/ml [MCF7]) for 24 hours. The resultant cells were analyzed by immunoblotting using antibodies specific to K8/K18, DR4, DR5, caspase-3, caspase-8, and glyceraldehyde 3-phosphate dehydrogenase (GAPDH). Caspase activation was identified by decrease in pro-enzyme form (ProC-8 and ProC-3). **(D-F)** Cells were treated as above and analyzed by flow cytometry after staining with Annexin-V-FITC and propidium iodide (PI).

To examine the effects of K8/K18 expression on apoptotic signaling, we transfected small interfering RNA (siRNA) against keratin 8 gene (*KRT8*) into K8/K18-expressing breast cancer cell lines (T47D, BT474, and MCF7). Immunoblot analysis confirmed effective K8 knockdown in targeted cell lines (Figure 2A-2C). As expected, there was a concomitant decrease in K18 protein levels, presumably due to spontaneous degradation as previously reported [23]. A scramble siRNA was used as a control. Interestingly, loss of K8/K18 pair resulted in a significant increase in DR5 protein levels while having little effect on DR4 protein expression (Figure 2A-2C). To determine whether this was a transcriptional effect, we measured DR4 and DR5 mRNA expression using a Qiagen apoptosis PCR gene array. The results revealed no difference in DR4 or DR5 mRNA expression levels between K8-deficient cells and their corresponding wildtype counterparts (data not shown). Hence, K8/K18 appears to selectively regulate DR5 protein expression through post-translational pathways; however, the precise underlying mechanisms remain to be defined. Following this observation, we treated the cells with TRAIL and measured caspase activation and apoptosis. Knockdown of K8 in the three cell lines enhanced TRAIL-induced activation of caspase-8 and caspase-3 (Figure 2A-2C), and subsequently sensitized the cells to TRAIL induced apoptosis which were otherwise resistant to TRAIL killing (Figure 2D-2F). These data suggest that K8/K18 selectively controls apoptosis signaling *via* DR5.

### Keratin 8/18 overexpression downregulates DR5 protein expression

**Figure 3:**
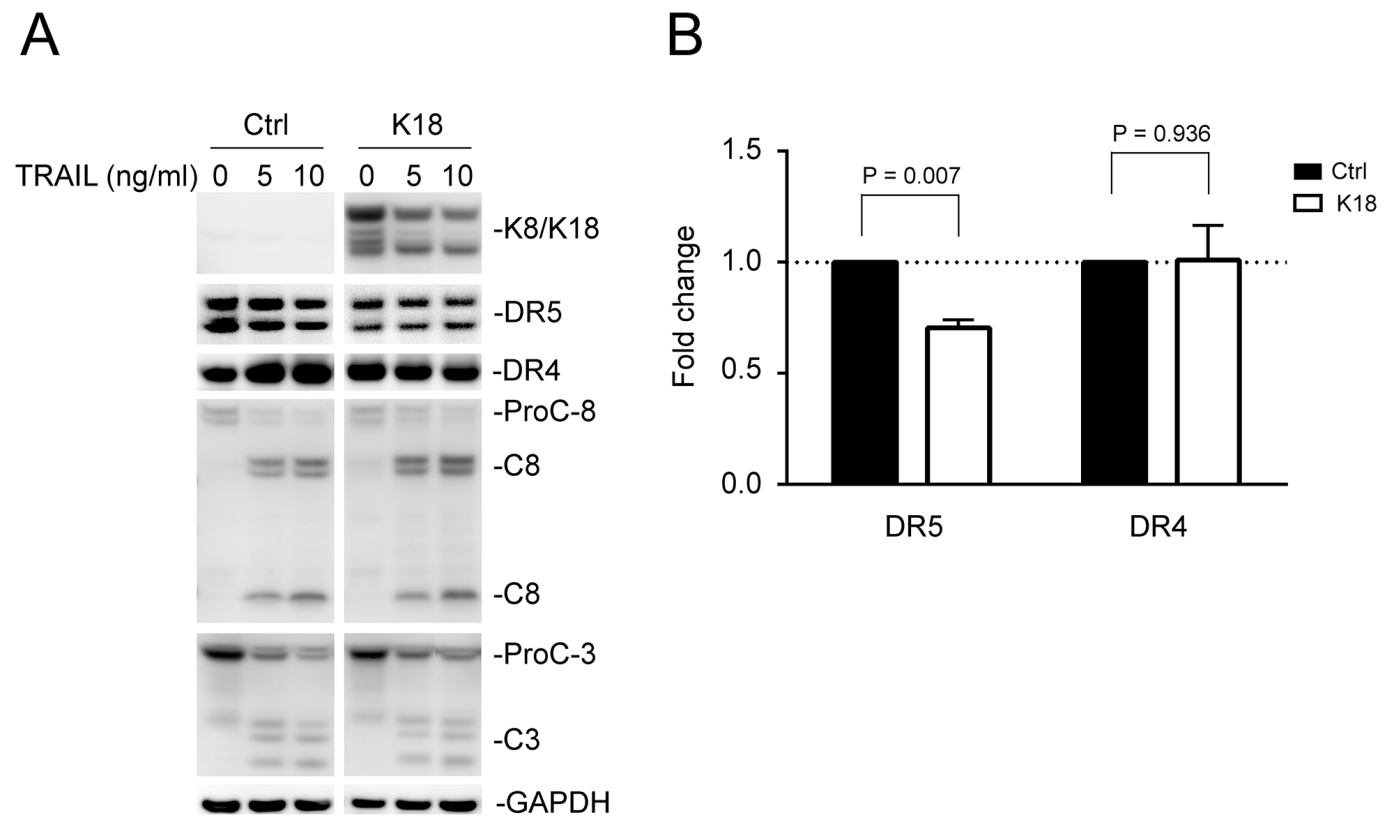
Keratin 8/18 overexpression downregulates total DR5 protein levels **(A)** MDA-MB-231 cells were transfected with a control plasmid or plasmid encoding human keratin 18 for 24 hours, followed by TRAIL stimulation (0, 5, 10 ng/ml) for 3 hours. The resultant cells were analyzed by immunoblotting using antibodies against K8/K18, DR5, DR4, caspase-8, caspase-3, and glyceraldehyde 3-phosphate dehydrogenase (GAPDH). Caspase activation is indicated by a decrease in pro-enzyme form (ProC-8 and ProC-3) and by the simultaneous appearance of cleaved fragments. Results are from non-adjoining lanes from the same gel. **(B)** Relative total DR5 and DR4 protein levels were quantified using densitometry analysis of immunoblots in A and were normalized to GAPDH loading controls. P-values were determined using a student’s *t*-test.

To test the effect of K8/K18 overexpression, MDA-MB-231 cells, which express low levels of endogenous K8/K18 (Figure 1A), were transiently transfected with a control cDNA plasmid or plasmid encoding human keratin 18. Immunoblot analysis revealed successful overexpression of both K8 and K18, which was accompanied by a significant decrease in total DR5 protein in target cells (Figures 3A and 3B). By contrast, DR4 expression levels were not affected by K18 transfection. As noted in Figure 3A, caspase activation showed no difference in TRAIL sensitivity between control cells and cells expressing K8/K18. This might be due to the existence of DR4, whose expression was not affected by K8/K18 overexpression. In support of this possibility, it is known that TRAIL can engage DR4 and/or DR5 to induce apoptosis.

### Keratin 8/18 physically interacts with DR5

**Figure 4:**
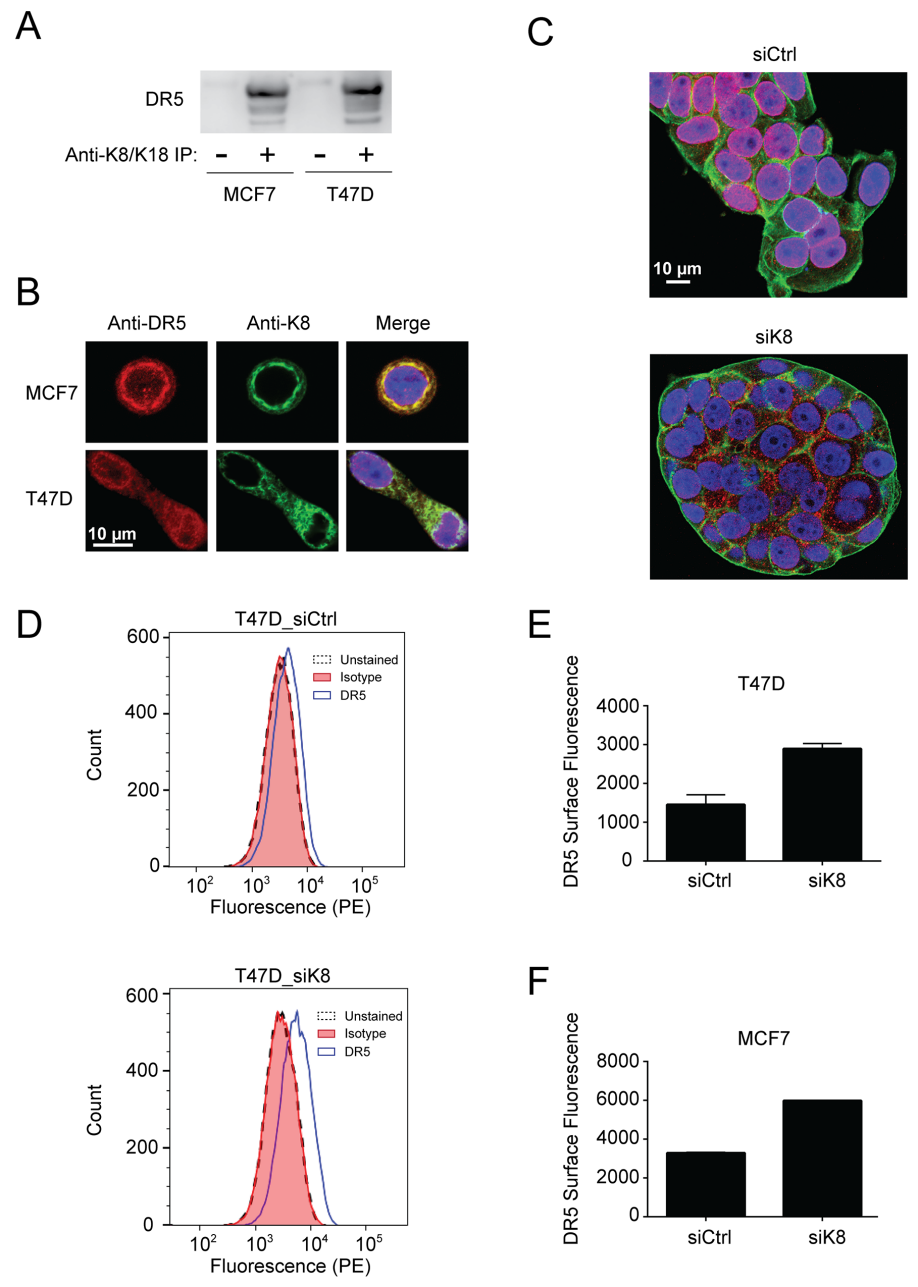
K8/K18 physically interacts with DR5 **(A)** Cell lysates from MCF7 and T47D cell lines were subjected to immunoprecipitation (IP) with an anti-K8/K18 antibody immobilized to protein A-agarose (lane 2 and 4) or with protein A-agarose alone (lane 1 and 3). IP samples were immunoblotted for DR5. **(B)** Cells were fixed, permeabilized, stained with anti-DR5 antibody (red) and anti-keratin 8 antibody (green), and analyzed by confocal microscopy. All images were acquired using a 40x objective lens. **(C)** MCF7 cells were transfected with control siRNA or siRNA against *KRT8* for 72 hr. The resultant cells were fixed, permeabilized, and stained with anti-DR5 antibody (red), DAPI (nuclei, blue), and fluorescent phalloidin 488 for actin visualization (green). All images were acquired using a 40x objective lens. **(D-F)** Flow cytometry analysis of cells transfected with control siRNA or siRNA against *KRT8*. The resultant cells were harvested using a non-enzymatic cell dissociation solution. The cell samples were incubated with PE conjugated anti-DR5 antibody (IgG2b) or its corresponding IgG2b isotype as a control. (D) Shown are representative histograms of unstained cells (black dashed line), cells stained with the isotype control (red fill), and cells stained with PE-anti-DR5 antibody (blue outline with no fill). The right shift represents the presence of DR5 on the cell surface membrane. Median PE-anti-DR5 antibody fluorescence intensity for cells transfected with control siRNA and siRNA targeting keratin 8 are shown after subtraction of isotype control median fluorescence intensity in two independent experiments for T47D cells (E) and MCF7 cells (F).

We next examined whether K8/K18 physically interacts with DR5 in breast cancer cells. MCF7 and T47D cell lysates were incubated with an anti-K8/K18 monoclonal antibody conjugated to protein A-agarose and isolated immunocomplexes were immunoblotted for DR5. Notably, the result showed a strong interaction between K8/K18 and DR5 (Figure 4A). Immunofluorescence staining coupled with confocal microscopy revealed that consistent with our previous observations [20-22], DR5 resided mainly in intracellular compartments including nuclear and perinuclear areas (Figure 4B). K8 was exclusively perinuclear and cytosolic, overlapping with DR5 at these locations (Figure 4B). Upon K8 knockdown, DR5 diffused from the nuclear and perinuclear areas (Figure 4C). Furthermore upon K8 knockdown, flow cytometry analysis demonstrated a significant increase in the expression levels of DR5 on the surface of targeted cells (Figure 4D-4F). By contrast, endogenous DR4 was not detected on the surface of control cells (MCF7 and T47D) and there was no apparent increase upon K8 knockdown (data not shown). Collectively, K8/K18 appears to physically interact with DR5 in breast cancer cells thereby regulating apoptotic signaling induced by TRAIL or receptor agonists.

## DISCUSSION

One of the key challenges in developing DR5-targeted therapies has been to identify biomarkers that might help predict tumor responsiveness. Here we identified keratin 8 and keratin 18 intermediate filament protein pair (K8/K18) as a functional regulator of apoptosis *via* DR5. Abundance of K8/K18 protein is correlated with TRAIL resistance in a panel of human breast cancer cell lines. Knockdown of K8 increased DR5 expression on the surface membrane and subsequently sensitized TRAIL-resistant cell lines to TRAIL induced apoptosis. These data warrant additional studies to evaluate K8/K18 as a potential biomarker of TRAIL sensitivity in primary breast tumors as well as other tumor types of epithelial origin.

**Figure 5:**
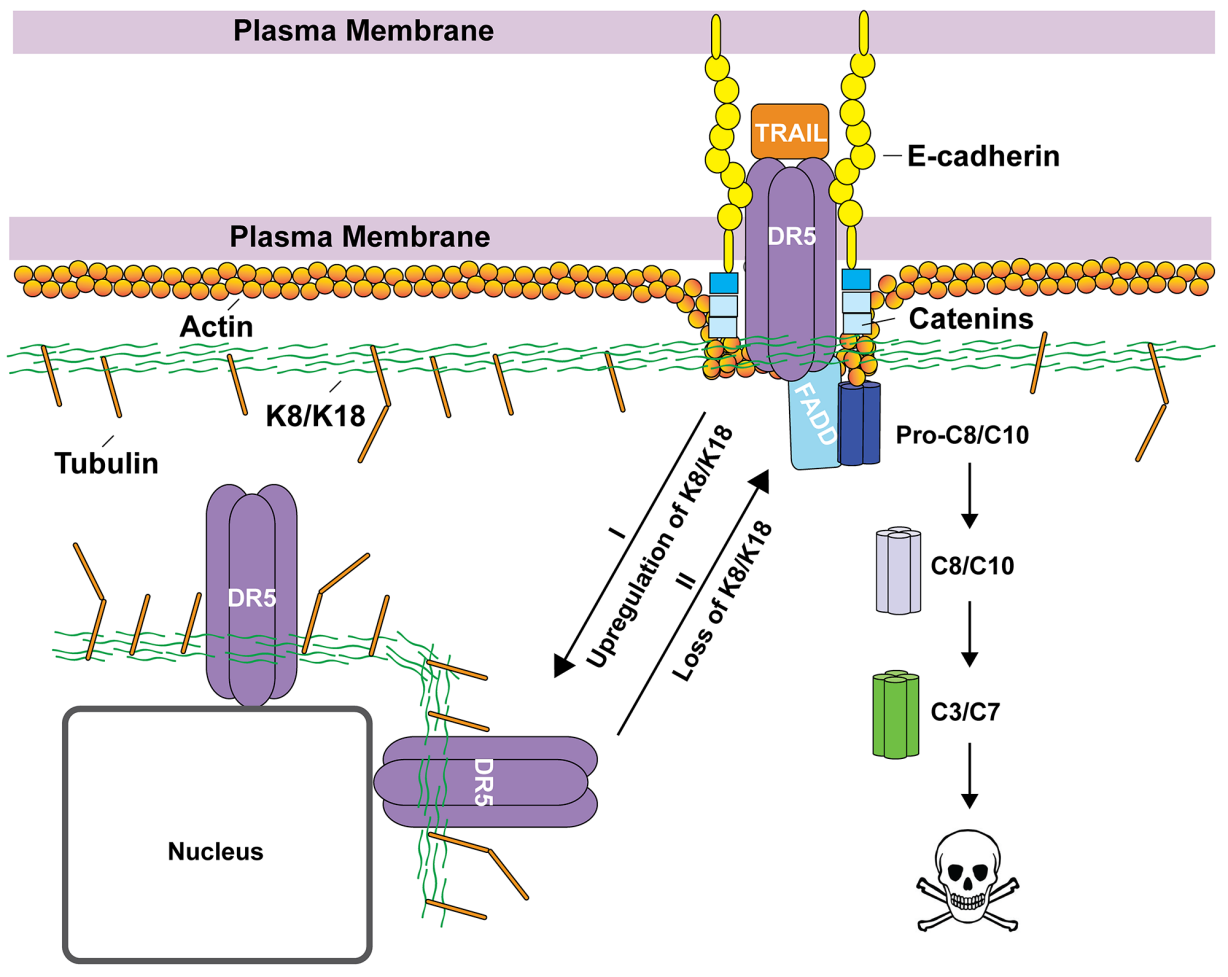
Proposed working model by which K8/K18 regulates apoptosis via DR5 K8/K18 proteins are found to be upregulated in TRAIL-resistant cancer cells. K8/K18 pair appears to physically interacts with DR5, restricting the receptor inside the cells (e.g., localized in nuclear and perinuclear compartments) (Route I). Loss of K8/K18 results in translocation of DR5 to the cell surface, thereby restoring apoptotic signaling via DR5 (Route II). Other cytoskeleton components (e.g., tubulin and actin) and interacting proteins (e.g., catenin and E-cadherin) may be integral in the regulation of death receptor mediated apoptosis.

Accumulating evidence shows that K8/K18 are not only markers of simple epithelial cells (e.g., mammary gland) but are also active regulators of cancer cell signaling [7-9]. The loss of K8/K18 expression in epithelial-mesenchymal transition (EMT) is linked to metastasis [24, 25]. On the other hand, K8/K18 overexpression was shown to render tumors resistant to chemotherapeutic agents [10-12] and to TNF/FasL death ligands [13-17]. Our data demonstrate that K8/K18 protects breast cancer cells from apoptosis *via* DR5 (Figure 5), which can provide tumor escape from immune surveillance. Selective knockdown of K8 effectively restored TRAIL sensitivity, which was at least partly through upregulation of DR5 on the surface of targeted cells (Figure 2 & 4). Consistent with this data, triple negative breast cancer cell lines, which are generally characterized by low K8/K18 expression, have been shown to be highly sensitive to TRAIL killing [26]. These data provide a rationale for therapeutic strategies to target DR5 for treatment of triple negative breast cancers (Figure 5).

As a death receptor, DR5 must be expressed on the surface membrane to interact with incoming death ligand (e.g., TRAIL). However, several reports revealed DR5 localization in nuclei and other intracellular compartments [27-29]. Importantly, surface deficiency of DR5 has been shown to suffice for cellular resistance to TRAIL or anti-DR5 agonistic antibodies [20]. Interestingly, knockdown of K8 selectively increased DR5 expression, but not DR4, at both the total protein level and at the surface of targeted cells (Figure 2 & 4). This was supported by flow cytometry and confocal imaging (Figure 4). The mechanisms by which K8/K18 regulate DR5 protein expression remain to be defined but may involve K8/K18-mediated protein degradation pathways. To support this notion, DR5 mRNA expression was not affected by K8 knockdown. A recent study showed that K8 promotes autophagic clearance of cargo proteins by facilitating autophagosome-lysosome fusion [30]. Our lab has previously found that DR5 can be sequestered into autophagosomes in some cancer cells, resulting in its deficiency on the cell surface [21]. It is possible that K8/K18 may control DR5 protein expression through modulation of autophagy pathways.

The cytoskeletal network can interact and regulate death receptors through multiple mechanisms (Figure 5). Our lab has recently found a similar role for tubulin proteins in regulating DR5 (unpublished results). Lu et al. (2014) showed that actin is required for proper DISC assembly and caspase activation upon TRAIL stimulation [31], where E-cadherin tethers ligand bound death receptors to the cytoskeleton through catenins (Figure 5). Our data show that K8/K18 directly affects DR5 subcellular localization in which loss of K8/K18 results in DR5 translocation to the plasma membrane. This K8/K18 receptor targeting modulation was previously observed for Fas trafficking [17]. These data suggest that the cytoskeleton network which is composed of K8/K18, actin, and tubulins may play an integral role in regulating apoptosis via death receptors.

## MATERIALS AND METHODS

### Cell lines and materials

The human breast cancer cell lines including AU565, BT474, MDA-MB-453, HCC1428, MDA-MB-361, T47D, MCF7, ZR751, HCC1500, HCC1937, HCC1954, MDA-MB-468, SKBR3, MDA-MB-157, MDA-MB-231, HCC38, HCC2157, and BT549 were purchased from American Type Culture Collection (ATCC). All cell lines were cultured per ATCC recommendation and were tested for the absence of mycoplasma contamination regularly. Recombinant human TRAIL protein, containing amino acids 114-281 of human TRAIL was produced by E. coli as homotrimers and was purchased from R&D Systems (375-TEC). Antibodies specific to human caspase-3 (8G10), caspase-8 (1C12), DR4 (D9S1R), and DR5 (D4E9) were purchased from Cell Signaling Technology. Keratin 8/18 antibodies were purchased from Cell Signaling (C51) and BioLegend (1E8). GAPDH antibody was purchased from Novus (2D4A7). Horseradish peroxidase–conjugated goat anti-rabbit IgG1 (sc-2054), goat anti-mouse IgG1 (sc-2969), and donkey anti-goat IgG (sc-2020) were purchased from Santa Cruz Biotechnology. Phycoerythrin (PE)-conjugated monoclonal antibodies to DR4 (FAB347P) and DR5 (FAB6311P) and corresponding IgG1 (IC002P) and IgG2b (IC0041P) controls were purchased from R&D Systems.

### mRNA expression

mRNA expression was determined using a RT^2^ Profiler Apoptosis PCR array through contract service with Qiagen.

### Small interference RNA silencing

Synthetic small interference RNA (siRNA) oligos specific to keratin 8 (GAAGCAACAUGGACAACAUTT) or validated nontargeting control siRNA was purchased from ThermoFisher Scientific. Cells were grown to 50-70% confluence and transfected with siRNA oligos for 72 hours using lipofectamine. When indicated, cells were incubated with rhTRAIL (100 or 150 ng/ml) for an additional 24 h. Cells were harvested using non-enzymatic cell dissociation solution, Cellstripper (Corning).

### Plasmid transfection

pCMV6-ENTRY cDNA plasmid encoding human keratin 18 and a control plasmid were purchased from Origene Technologies. MDA-MB-231 cells were grown in 6-well plates to 70 % confluence and were transfected with plasmids using X-tremeGENE HP DNA transfection reagent (Millipore Sigma) for a duration of 24 h. As needed, transfected cells were incubated with rhTRAIL (0, 5, and 10 ng/ml) for 3 hours followed by immunoblotting for caspase activation.

### Immunoblot analysis

Cells were lysed using RIPA buffer [0.5 M Tris-HCl (pH 7.4), 1.5 M NaCl, 2.5% deoxycholic acid, 10% NP-40, 10 mM EDTA, and protease inhibitor cocktail] (Millipore). Cellular debris was removed by centrifugation and total protein amount was determined by BCA assay (ThermoFisher Scientific). Equal amount of total protein (20-30 μg) was resolved by SDS-PAGE using NuPAGE 4–12% gradient Bis-Tris gels (Life Technologies) and transferred to PVDF membranes. Membranes were blotted with appropriate antibody and visualized using Immobilon Western Chemiluminescent HRP Substrate (Millipore) and an ImageQuant LAS 4000 imager (GE). Protein expression levels were quantified by densitometry analysis of immunoblots using the imager built-in software and were normalized to the corresponding actin or GAPDH loading controls. P-values were calculated using student’s *t*-test.

### Immunoprecipitation

Cell pellets were lysed in RIPA buffer. In 1 ml total volume, 1 mg of protein lysate was incubated with 6 μg of anti-K8/K18 antibody, purchased from BioLegend (1E8) for 4 h at 4 °C. 60 μl of protein A-agarose slurry (Santa Cruz) was then added and further incubated overnight at 4 °C. The beads were separated from the lysate using centrifugation and were washed 5 times using lysis buffer. After the last wash beads were treated with 1 X LDS sample buffer and heated at 90 °C for 10 minutes. IP samples were analyzed by SDS-PAGE and immunoblot analysis as previously described.

### Flow cytometry

To quantify apoptosis, cells were labeled with Annexin V-FITC and propidium iodide (PI) using an eBioscience Apoptosis kit (88-8007-72). Labeled cells were analyzed using BD Accuri C6 flow cytometer (BD). Flow cytometry analysis of surface receptors was performed as previously described. Briefly, cells were harvested using non-enzymatic cell dissociation solution Cellstripper (Corning). Cell pellets were blocked in PBS buffer containing 5% normal goat serum and 1 % bovine serum albumin on ice for 20 minutes. Cells were then incubated on ice for 45 minutes in the dark with phycoerythrin (PE)-conjugated anti-DR4 or anti-DR5 monoclonal antibodies or corresponding PE-conjugated isotypes. Cells were subsequently washed twice with PBS buffer, resuspended in PBS buffer containing 1 % bovine serum albumin and analyzed.

### Confocal microscopy

Cells were seeded onto sterilized coverslips coated with poly-L-lysine. Attached cells were fixed with 4 % paraformaldehyde in PBS buffer for 15 minutes and were permeabilized using PBS buffer containing 0.5 % triton X-100 for 15 minutes. Cells were then blocked with PBS buffer containing 3 % BSA and were labeled with specific primary antibodies, anti-keratin 8 rabbit mAb (Abcam, ab53280) and anti-DR5 mouse mAb (Santa Cruz, 166624). Alexa Fluor 594 goat anti-mouse and Alexa 488 goat anti-rabbit antibodies were used as secondary antibodies for detection (ThermoFisher Scientific). Nuclei were stained with DAPI and actin was stained with phalloidin conjugated to Alexa 488 dye. Confocal images were captured using an LSM 510 Meta Confocal Microscope attached to an Axiovert 200 Inverted Microscope (Carl Zeiss). An oil immersion 40x objective lens was used for all optical imaging. Laser lines of 405, 488, and 594 were used to detect DAPI, Alexa 488, and Alexa 594 respectively.
